# Ethnobotanical Study of Traditional Medicinal Plants Used by the Local People in Mekdela Woreda, South Wollo Zone, Ethiopia

**DOI:** 10.1155/sci5/8875307

**Published:** 2025-06-16

**Authors:** Abdurahman Wondimnew, Yalew Yiblet, Anchiye Getachew, Tegegn Muche, Habitamu Amsalu

**Affiliations:** Department of Biology College of Natural and Computational Science, Mekdela Amba University, P.O. Box 32, Tulu Awuliya, Ethiopia

**Keywords:** ailment, folk medicine, home garden, local knowledge, remedies

## Abstract

Folk medicine has long been the most popular availability and hospitalization option for those who are unable to afford modern medications in the primary healthcare system. But migration, industrialization, and other factors are contributing to the disappearance of indigenous herbal medicine insight. The aim of this study was to point out, register, and investigate the traditional applications of botanical medicines and the traditional ecological knowledge in the Mekdela woreda, South Wollo Zone. By applying the Cochran formula, 384 participants in total (256 men and 129 women) were chosen. And from a total of 32 kebeles, 9 kebeles were chosen using selective sampling, and the chosen group of households was made using a stratifying sampling method followed by random sampling techniques. And, 74 kinds of plants, categorized into 42 genera and 63 families, were gathered from the research area. At the investigation site, these varieties of plants were recommended to treat 44 illnesses (16 in animals and 28 in humans). A total of 54% of plants used for medicinal purposes were common, 23% were medium, and 23% were rare. *Allium sativum* was discovered to possess the highest informant consensus. The majority plants that were best at powdering, pulverizing, and mixing with water were chosen to make the treatments. Leaves and roots accounted for 49% and 21% of the remedies, respectively. Oral administration is used in 67% of applications and is an extremely frequently employed technique of administration. The locals used cups (sini), glasses (birchiko), spoons (mankia) to determine the dosage of remedies.

## 1. Introduction

The term “traditional medicine” describes methods, approaches, and understanding of the treatment, diagnosis, prevention, and healthcare practice of medicines that are driven by plants, animals, and minerals, training, spiritual therapy, and biotechnology [1]. Traditional medicine remains the main means of treating human and animal diseases and is essential to fulfilling the fundamental requirements for the health needs of developing countries. The scientific study of plant materials utilized in native cultures such as ceremonies, cuisine, biomedical and miracles, architecture, wood, musical equipment and domestic tools, insecticides, clothing, and shelters is known as ethnobotany [2].

Traditional medicines are the most crucial sources of medicine for approximately 80% of the total number of people in Ethiopia. For example, 85% of the majority of people on the planet utilize herbal medicines to prevent and treat diseases, and demand for these products in both developed and developing nations has been expanded [3, 4]. Ethiopians speak a wide variety of languages and have many cultures and religious beliefs, contributing to a very different traditional knowledge base and methods of managing and conserving the country's plant resources [3, 4]. Traditional medicine is mainly a locally accessible medical plant and is an essential component of the medical systems, among rural Amhara communities in Mekdela district, as it would elsewhere. However, due to the widespread ecological deterioration that seems to have occurred in the worst way in this region, locally available medicinal plants are increasingly scarce, jeopardizing this knowledge and practices. There are currently no details regarding the traditional medicines' use, potential harm, or preservation at the study site.

Ethnobotanical studies focus on the relationships between people and plants, particularly how local communities utilize plant resources for medicinal purposes. These studies are crucial for several reasons. For instance, ethnobotanical studies are essential for preserving indigenous knowledge, conserving biodiversity, facilitating drug discovery, understanding cultural significance, enhancing healthcare accessibility, and promoting sustainable practices within local communities [3, 4]. Many gaps remain in the current body of research, especially with regard to the thorough understanding and preservation of this knowledge. These include limited geographic coverage, inadequate documentation of indigenous knowledge, threats to medicinal plant resources, gaps in integration with modern healthcare systems, and a lack of standardization in research methodologies. Ethnobotanical studies are essential for recording the traditions and local knowledge surrounding medicinal plants used by local communities [4, 5].

For this reason, it is critical to record traditional applications of herbal remedies with the aim to protect native traditions. The objectives with this investigation include documenting plant species used for medicinal purposes, preserving traditional knowledge about their uses and preparations, assessing cultural practices surrounding these plants, evaluating their efficacy through community consensus, identifying conservation needs for threatened species, and incorporating indigenous treatments into Mekdela district, South Wollo Zone, Amhara Region, Ethiopia's official health services.

## 2. Materials and Methods

### 2.1. Description of the Study Area

**Figure 1:**
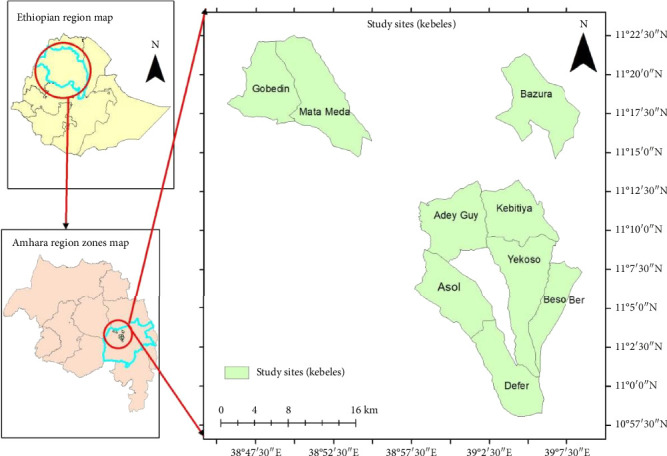
Administrative map of Mekdela Woreda (source; Google Earth [2024]).

**Figure 2:**
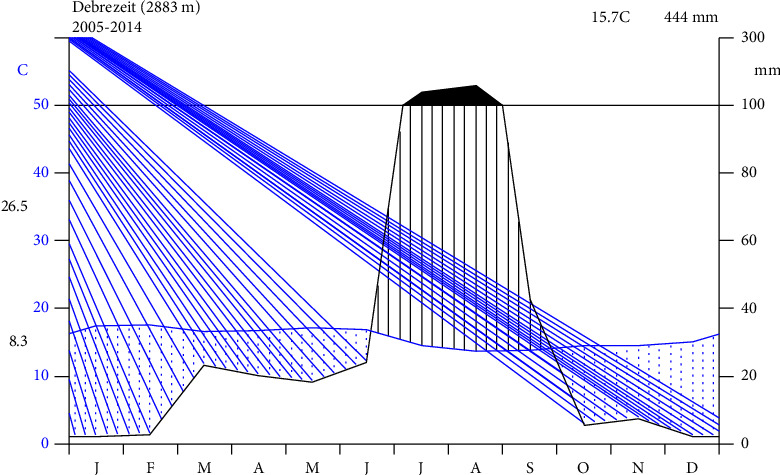
Average monthly temperature and mean monthly rainfall of Mekdela Woreda (source: National Meteorology Agency of Ethiopia Kombolcha branch [2024]).

The research was carried out in Mekdela district, South Wollo Zone, Amhara region, Ethiopia. It is situated 552 km away from Addis Ababa, 152 km from Dessie (Figure 1). The study areas extend between 11.83–110.93E longitude and 120.21–120.4N latitude [6]. It has wide altitudinal variation above sea level, 2055–3315 m.a.s.l. [7]. The study area receives seasonal and moderate amount of rainfall (Figure 2).

### 2.2. Study Design

Traditional systems of medicine utilized by the community to treat various illnesses at the study site were assessed using a community-based cross-sectional research methodology.

### 2.3. Data Sources

Ethnobotanical methods are often used to compile data on the administration and understanding of plants for healing utilized by the local community [8]. Primary and secondary sources of data have been used.

### 2.4. Choosing Research Areas and Respondents

The following presumptions were used to represent the population depending on the predominance of knowledge, perception, and discipline. It is preferable to predict the following equations to determine the large enough sample size based on the proportion with an estimated 95% level of certainty. These were obtained with a *Z*-value of 1.96 and a marginal error of 5%. Research papers or other scientific papers are not available in the study area. So, a *p*-value of 50% was taken. Therefore, the sample size will be calculated as follows.

Such that *Z* is generally accepted typical value that corresponds to the desired level and *n* is the number of samples. Of confidence, *p* is an estimate of the proportion of the population and the variable of interest and the greatest permitted inaccuracy is denoted by *E* [5]. Hence,

(1) $$\begin{array}{c} n=0.5\left(1-0.5\right)\left(\frac{1.96}{0.05}\right)2\\ =0.5\times 0.5\left(39.2\right)2=384.5∼\;385. \end{array}$$

And from the total of 32 kebeles, nine kebeles have been picked in accordance with the agroecology in the district from the Dega (Yekosso, Bessober, Defer). Woyinadega (Gobedin, Adyguya, Kibtia) and Kolla agroecology (Matameda, Assol, and Bazura) were used as samples using purposive sampling methods, and the group of households was selected using stratified sampling techniques, using types of probability sampling methods by creating strata in gender, job profile, age, and education and by following simple random sampling.

### 2.5. Compilation of Ethnobotanical Information

Conducting fieldwork for gathering indigenous plant insights took place between January 20 and May 30, 2023. The main techniques used to gather data for the study were qualitative methods like an interview and focus group discussion and qualitative methods like survey and questionnaires, and field observations were used.

### 2.6. Collection and Identification of Plant Specimens

In the field, preliminary identification was completed, and the specimens obtained were transferred to the Ethiopian University of Addis Ababa's National Herbarium. After they have been collected using Geolocation information and names from the community, pressed down, and preserved, accompanied specimens were prepared for final conclusions and validation of Ethiopian and Eritrean flora applying taxonomy keys.

### 2.7. Data Analysis

#### 2.7.1. Descriptive Statistics

The information on medicinal plants and related knowledge was analyzed and summarized using statistical techniques that were descriptive, including percent, rate, and correlation.

#### 2.7.2. Informant Consensus

The respondents were approached for the same information at least a couple of times, and the validity of the information was confirmed and documented in order to assess the dependability of the information captured during the discussion. So that informant consensus factor (ICF) would be determined by dividing the number of applications cited for each classification by one, using the number of use references for each category (nur) minus the number of species utilized (nt) for those categories. ICF = nur − nt/nur − 1.

#### 2.7.3. Preference Ranking

The five indigenous healers typically treat stomach problems with the most essential healing plants. To find the most popular species of herbal remedies for treating stomachaches, eight respondents were chosen. Each respondent was given five plants with medicinal properties that were said to cure this illness; each leaf of the plant was given a paper-tagged name. The informants were then asked to rank the plants in order of preference, giving the most favored plant species a value of (5) and the least favored plant a value of (1).

#### 2.7.4. Paired Comparison

A collection of assortments from the selected items is generated, encompassing every possible mix, and the order of pairings is randomized. These pairs are then presented to selected informants, who provide their responses and summarize the overall value. Eight informants in this study reported on the effectiveness and popularity of six healthful plant species used to treat snake nips leading to the creation of a rating based on their insights.

#### 2.7.5. Direct Matrix Ranking

The purpose is to evaluate a species' multifunctional usage and connect it to the degree of its primacy in contrast to its use. Out of all the herbal remedies, eight multifunctional plant species were chosen based on the information obtained from sources. Medicine, livestock feed, food, fuel, building, charcoal, fencing purposes, and the manufacture of furniture are among the eight use-values. In order to carry out this exercise, fifteen key informants were selected, and each one was requested to provide use-values: five for best, four for very good, three for good, two for less used, one for least used, and zero for not utilized. The eight multifunctional therapeutic plant species were therefore rated by each key source, and the mean value of each species' use-diversity was calculated, added together, and ordered.

#### 2.7.6. Fidelity Level Index

The significance of a species for a certain function within a given cultural community is measured by the fidelity level index. The potential effectiveness of any plant remedies cannot be determined just by confirmation or consensus. As a result, the presence of a certain plant and illness have an impact on the informant choice in addition to efficacy. With Np being the number of informants who reported using a plant species to treat a specific ailment and *N* being the number of sources who used the plant as medicine to cure any given sickness, the fidelity level index Fl = Np/(*N* × 100).

## 3. Results and Discussion

### 3.1. Characteristics of the Respondents

**Figure 3:**
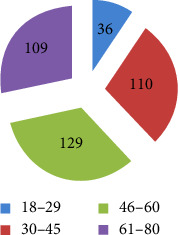
Age classes of the respondent.

**Figure 4:**
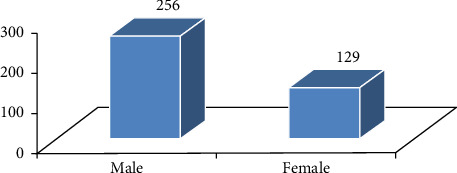
Sex ratio of the respondents.

**Figure 5:**
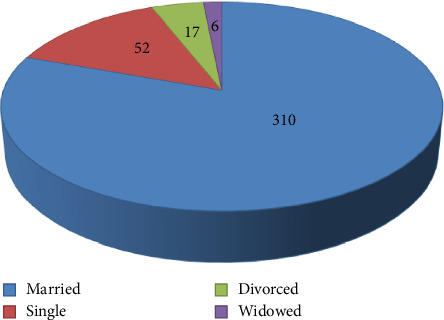
Marital statuses of respondents.

**Figure 6:**
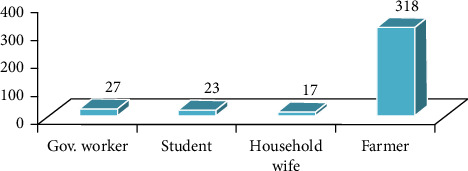
Occupation of the respondents.

**Figure 7:**
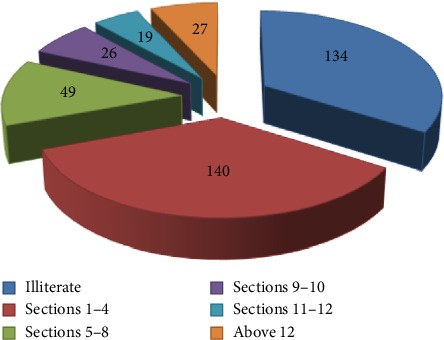
Educational background of the respondents.

**Figure 8:**
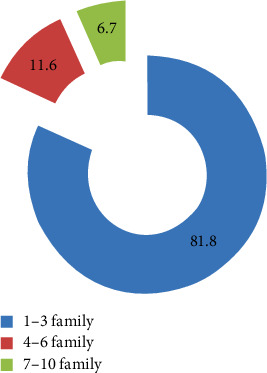
Family number of the respondent.

Information on the background of the participants was gathered to have a clear picture of the respondents. All the respondents were households, which were found in Mekdela woreda. The four classes of age characteristics show that 43 (11%) of the respondents were aged 18–29, 105 (27%) were aged 30–45, 136 (35.3%) were aged 46–60, and 102 (26.4%) were aged 61 and above (Figure 3). Regarding sexes, 255 (66.2%) of households were males and only 130 (33.7%) households were females (Figure 4). Most were married; only 21 (5.45%) were single (Figure 5). Based on occupation, 81% are farmers, 15.9% governmental workers, and 3% students (Figure 6). Regarding educational level, 73 (18.9%) are illiterate and 134 (34%) are grades 1–4; 106 (27%) are 5–8; 32 (10%), 9-10; 14 (3.6%), 11-12; and 26 (6.7%) above 12 (Figure 7). As far as family members, 127 (32%), 1–3; 198 (51.4%), 4–6; and 60 (16%) households have 7–10 family members (Figure 8). From the background information of the respondents, all are Amharic speakers and the majority of households were farmers.

### 3.2. Composition and Habit of Medicinal Plants

**Figure 9:**
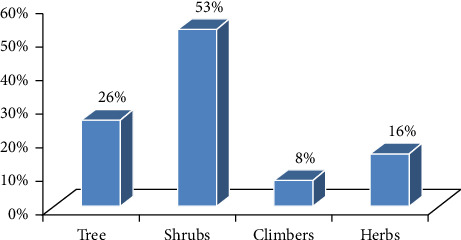
Medicinal plant habits in the research area.

In the field of research area, 74 species of medicinal plants were identified, representing 42 families and 63 generations. The proportion of shrubs, trees, herbs, and climbers in the forest is as follows: 38 (53%), 17 (26%), 14 (16%), and 5 (8%), respectively (Figure 9). Based on current research, shrubs are the most prevalent habit of using medicinal plants in the area under study, with trees and herbs, which come second. This might be the result of these species' high abundance and ease of acquisition. According to earlier reports, Ethiopia is home to a comparatively large number of herbs and shrubs with medicinal uses [9–11]. Besides this, this study demonstrated that similar proportions of medicinal plant growth forms are employed in therapeutic settings, potentially lowering the risk of medicinal plant use associated with restricted collection practices.

### 3.3. Medicinal Plant Record in the Research Area

There are 74 species in all, representing 63 genera and 42 distinct plant families. Of these, the Fabaceae and Lamiaceae families have the most, with five species each, followed by the Rutaceae, Oleaceae, Anacardiaceae, and Polygonaceae families with four species (6%); the Euphorbiaceae, Apiaceae, Verbanaceae, Rhamnaceae, and Celastraceae families each having two species (4.7%); whereas the remaining families each had two species (5.5%). As a result, there was a consensus [3, 12]; it is true that the Lamiaceae family is among the largest in the Ethiopian and Eritrean flora and that both Fabaceae and Lamiaceae contain more medicinal plants than any other family (Book of Ethiopia and Eritrean) (Table 1).

#### 3.3.1. States of Indigenous Therapeutic Plants

**Figure 10:**
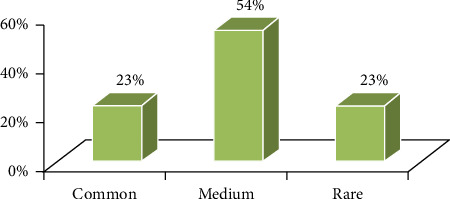
State of plant remedies in the research area.

The state of every herbal remedy was noted as common, medium, and rare according to healer remarks made throughout the semistructured interview and group discussion. From 74 indigenous therapeutic plants, 16 (23%) were noted as rare in the study area. This is due to the truth that most of these medicinal plants were distributed in the wild vegetation and hence the existence of pressure on wild plant remedies in the research area; 42 (54%) of plant remedies were recorded as common and 16 (23%) as medium (Figure 10). The later proportions of medicinal plant species are grown in a home garden and farm lands by the local people.

In frequent causes, plants in home garden were planted by older persons in the research field for the purpose of food and medicinal purpose. This is consistent with the finding of [13]; watering, trimming, fencing, and tree planting are all part of maintaining a home garden. Most elderly people spend the majority of their time in maintaining their home gardens. This is also in agreement with the finding of [3].

### 3.4. Useful Therapeutic Plants in Home Gardens

**Figure 11:**
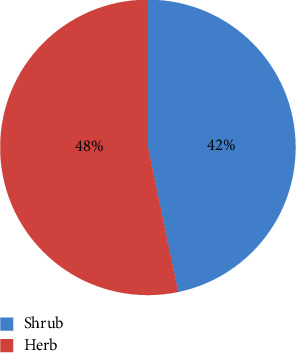
Life forms of medicinal plants in home garden.

Many home lawns are used to cultivate various inhabitants' use of herbal remedies of the research field. In each instance, perennial plants were planted in the garden areas outside the house (next to the fence), such as *coffee, Arabica, carica papaya, Eucalyptus globulus, phytolacca dodecandra*, whereas the ones nearer the house were set aside for annual plants like *Ocimum lamiifolium, Ruta chalepensis, Artemisia abyssinica, Allium sativum,* and *lepidium sativum*, which were cultivated with three species each; Solanaceae, Rutaceae, and Cucurbitaceae lead the other families based on the species structure. The Asteraceae and Brassicaceae come second and third, respectively, with two species, along with the other families, which have only one species each. In relation to life forms, among the 29 “home garden” plant species, 16 (48%) of the plant species are herbs, and 13 (42%) of the plant species are shrubs (Figure 11).

According to the current research, a significant number of herbs and shrubs with potential medical uses have also been documented in Ethiopia in the past [9–11]. Additionally, this study showed that comparable percentages of medicinal plant growth forms are employed in medicinal practices, which may help to slow the rate at which medicinal plants with limited habit collection become threatened.

**Figure 12:**
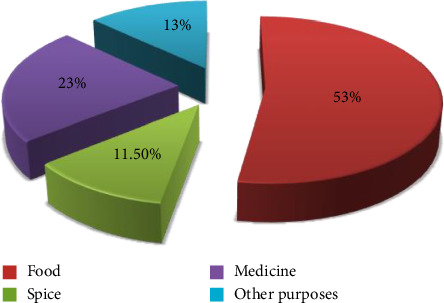
General functions of plants in home gardens.

The study also revealed that the crops grown in home gardens serve a number of purposes for the local population.  Table 2 demonstrates that approximately 14 species (53%) are connected to the food service industry, while 6 species (23%) are utilized in medicine. Three (11%) were used as spices, and four (13.5%) were used for other things like fences, household materials, and cash income sources. This result is consistent with the definition of a home garden provided by [5, 14], which highlighted that the primary objective of a home garden is to provide quick and simple access to food (Figure 12).

**Figure 13:**
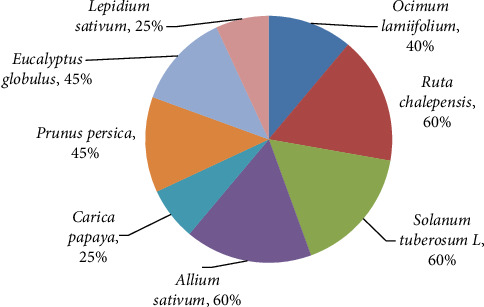
Plants that grow in home gardens with the highest relative frequency of occurrence.

The local aboriginal population is extremely aware of the crops in their home gardens. The majority of home garden or “guaro” owners recommend having food in their home garden or “guaro” throughout the year. They claim that a home garden or “guaro” is more beneficial and fruitful than their large agricultural fields. According to [15], which is consistent with the findings of the research, in Ethiopia, home gardens are said to be as old as agriculture (Figure 13).

### 3.5. Parts of the Plants Used as Herbal Medicine and Their Formulation

**Figure 14:**
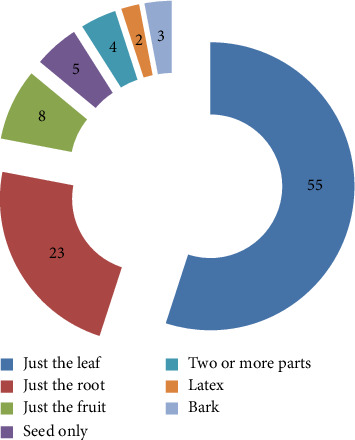
Plant components in preparation of remedies.

Local residents of the research field collect different plant components to prepare traditional medical applications. Leaves (51.8%) and roots (28.1%) had been the plant components that were most frequently used to make cures (Figure 14); this is consistent with the findings of other researchers [16]. This is due to easy anticipation and the inclusion of additional bioactive components in the leaves. It has also been established that leaves are mainly used for their potency and rapid regeneration [17, 18].

Studies conducted previously in Ethiopia have also demonstrated that the leaves and roots of plants are frequently utilized to treat a range of medical conditions [13]. To create a specific remedy, the medicinal plants were either used singly or in combination with multiple species. The synergistic effect could explain the use of various plants to cure a specific illness.

### 3.6. Formulation of Herbal Medication

**Figure 15:**
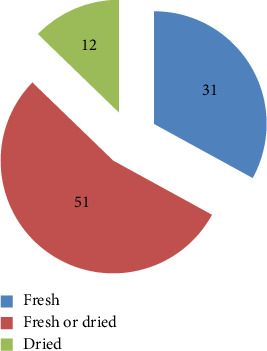
State of the plants used to make medicinal materials.

Informants who provided information about the preparation of medicine mentioned a variety of abilities related to preparing herbal remedies. These include the type of plant material used (fresh or dry), its composition (single or combination), and its preparation techniques. The findings indicated that 56.71% were used to make a variety of medicines, and roughly 43.29% were made from a combination of plant species. The result is consistent with the works by [19], which reported that combined plant materials have a high proportion of herbal preparations, and is in line with the findings of [20], which said that formulations made from just one plant were significant. In order to prepare remedies for their patients, herbalists typically use fresh specimens from easily accessible plants [19, 21]. This may be primarily because fresh medicinal parts of the plant are more effective for the treatment because they do not lose their contents before being used, unlike dried ones [12]. The results of a large portion of the plant parts utilized (58%) were either fresh or dried, according to the nature of the parts (Figure 15). It has been noted that a significant amount of combined plant materials are used in herbal preparations. In accordance with the findings of [22, 23], the majority of informant participants in discussion forums reported that they had used a variety of methods, such as chopping, and to preserve the plant material they were unable to locate during the dry season, they might either hang the entire plant material in the dining room or use powder.

**Figure 16:**
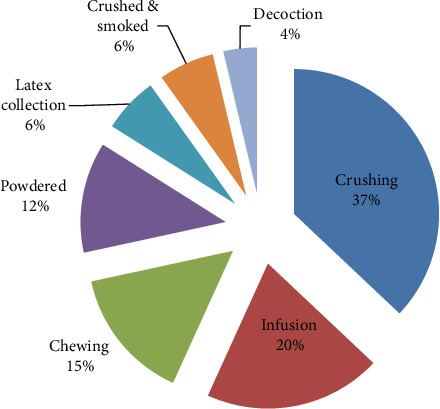
Way of preparing remedies in the study area.

Figure 16: Displays the outcomes of the different preparation methods. Crushing was one of the most common techniques (35.13%).

### 3.7. Mode of Application of Plant Remedies

**Figure 17:**
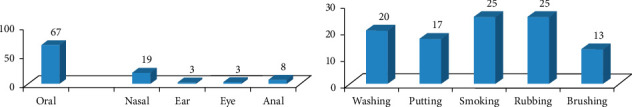
(a, b) The number of uses and mode of administration of natural remedies in the research area. (a) Internal routes of administration. (b) External routes of administration.

**Figure 18:**
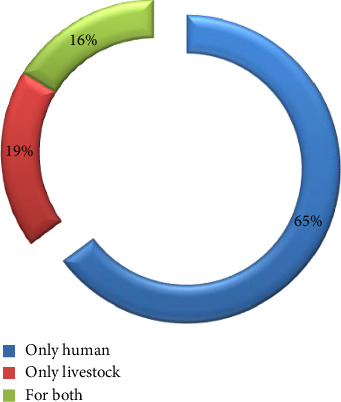
Percentages of indigenous medications used for curing illnesses in humans and animals.

Routes of administration in the research field have been either treated with topical medication (67%) or internally (59.5%), with oral utilization being the most common method (40.5%) and 25% of which smoke inhalation, and applying medication topically is the main (Figure 17). This could be due to the prepared medications responding more quickly to the metabolism of infections and having a greater curative effect when administered orally and topically [24, 25]. The existence of widely dispersed internal diseases in the research field is the other factor. The finding is consistent with some earlier reports [26–28] and research that also indicated that one of the most popular methods of administration was oral (Figure 18).

### 3.8. Important Indigenous Plants for Therapy in the Research Site

#### 3.8.1. Priority Ranking

The present finding revealed that the priority ranking of five therapeutic plants that are recommended to heal stomachaches with the highest informant consensus and it was evaluated by eight respondents according to their opinions and order of priority. *Ruta chalepensis* was ranked first, followed by *Allium sativum* (Table 1). This suggests that the indigenous people have used life experience to ascertain which plants work best for a certain condition.

#### 3.8.2. Paired Comparisons

Eight respondents were used to compare six of them in pairs in order to determine their grades. As a result, *Rumex nepalensis* was in second place, after *Calpurnia aurea* (Table 2). This finding suggests that *Calpurnia aurea* is preferred over other plant species used in the region for wound care. Furthermore, in the study area, the outcome may serve as evidence of the effectiveness of these two plant species in treating wounds.

#### 3.8.3. ICF

ICF was computed on the seven identified ailments. The greatest ICF value, 0.75, was on respiratory organs and throat, subsequent with abdominal and gastrointestinal problems of 0.57. This indicates there is homogeneity between information provided by informants and the quantity of sources mentioning a specific species or that the participants have a high degree of acceptance, while in the last one 0.28 were sexual and delivery problems. This indicates there is no homogeneity between the information provided and informants (Table 3).

#### 3.8.4. Fidelity Level

It was calculated on ailments that are frequently caused by informants. These ailments include stomachache, evil eye, bronchi, common cold, acute sickness, and wounds. To treat their common ailments, the indigenous therapists utilized important therapeutic plant species and their traditional knowledge was found for this ailment. The fidelity level of *Carissa spinarum* and *Clutia abyssinica* for evil eye was scored as 100, while it was 0.88 for *Ruta chalepensis* for acute sickness and *Rumex nepalensis* for stomachache (Table 4), which is calculated by the formula of index of fidelity level: Np is the percentage of participants who reported using a plant species to cure a specific ailment, and *N* is the amount of participants who used the plant as medication to heal any given condition. Fl = Np/(*N* × 100).

## 4. Ailments Cured in the Study Area

According to the current study, herbal medicines can treat 44 different kinds of health issues (Table 5), including 16 in livestock (Table 6) and 28 in humans. Of the 74 medicinal plants identified, 48 were employed exclusively to care for human health issues, 14 to cure livestock-specific conditions, and 12 to heal conditions that affect humans and livestock (Figure 18).

Forty-eight (65%) of the species of medicinal plants are known to be applied to the treatment of illnesses in humans (Table 7). Similarly, among medicinal plant species, 14 (19%) are reported to heal human and animal illnesses, while 12 (16%) were utilized for curing cattle illnesses.

### 4.1. Traditional Indigenous Knowledge of Medicinal Plants in Relation to Age

Most research indicates that age increases traditional medical knowledge. This finding agrees with the works of [10, 29–31]. Most studies show that traditional medical knowledge increases with age. The more people age, the more knowledgeable they are about medicinal plants, according to an analysis of the *t*-test for population correlation coefficient at the 5% level of significance and 1 degree of freedom (Df = *n* − 2 where *n* = 4) (Table 8). It is in the crucial area where there are sufficient data to support the thought that understanding plants for medicine and age are highly correlated [32, 33].

Take note of the above table, which shows that the t-critical value is greater than the *t*-calculated value and that the greater the medicinal plant knowledge, the greater the *t*-calculated value is. The *t*-critical value is 1.46 for the two degree of freedom and the total population correlation value (*p*), and 2.92 at the 5% level of relevance (Df = *n* − 2 where *n* = 4).

### 4.2. Risk to Therapeutic Plants and Associated Knowledge

#### 4.2.1. Risk to Therapeutic Plants

The depletion of native knowledge and therapeutic plants has been linked to a number of issues, including commercial overharvesting, abusive cutting practices, habitat loss from deforestation, and farming operations (Table 9). Therefore, therapeutic plants and plant species in general were seriously threatened by the demand for agricultural land and population pressure. In [34], it was mentioned how devastation affects therapeutic. Harvesting therapeutic plants is a consideration, but it is not as significant as the other elements as it puts them in danger as well [35]. The tragedy in the field of research is that understanding of the breadth and depth of medicinal plants is progressively declining due to their secrets, the unwillingness of young generations to acquire knowledge the impact of contemporary schooling, and religious and consciousness factors, all of which lead to a gradual extinction concerning traditional knowledge toward therapeutic herbs.

Plant destruction of construction materials, firewood, and grazing, browsing, and other purposes is causing the world to lose plants every minute [27]. In addition to certain natural factors, this frequent decline of plants was caused by human causes, genetic diversity, and endangered human survival by eroding some wild genes for life-saving medicinal plants [18]. Indigenous knowledge about plants and the benefits that can be obtained from them is linked to the disappearance of medicinal plants.

## 5. Conclusion

Seventy-four kinds of therapeutic plants were found and gathered, including 11 species found in both outside and domestic gardens. Specifically, 20 species were located in home gardens, while 43 were found in wild settings. Local healers reported the ability to treat a diverse range of illnesses, including rabies, hepatitis, ascaris, issues related to evil spirits, the ethos of the dead, the evil eye, and human-induced poison. In total, 44 illnesses were documented as being cured with traditional therapeutic plants, comprising 28 for humans and 16 for livestock. Among the 74 species, 48 are utilized for human ailments, 14 for livestock, and 12 for both. The predominant method of treatment is oral administration, with leaves being the most commonly used plant part, as they are perceived to be less harmful to the mother tree. However, the study area faces threats to its medicinal plants due to agricultural development, modernization, cultural shifts, and increased business activities.

One main contribution is documenting medicinal plant diversity. For example, a study in Mekdela district found 74 types of plants used for treating illnesses in humans and livestock. This documentation highlights the variety of available plants and their potential health benefits, serving as a basis for further research. These studies benefit health care, biodiversity conservation, cultural heritage, and economic development. These studies also help preserve indigenous knowledge that may be at risk due to modernization. By recording how local communities use these plants, ethnobotanical research protects valuable traditional practices. Traditional healers share important information about treating diseases with specific plants.

The study highlights valuable insights from ethnobotanical research in Mekdela district, Ethiopia, showcasing the interaction between local communities and their environment. It emphasizes the significance of indigenous knowledge in biodiversity conservation, sustainable resource management, and cultural heritage preservation. Local populations possess extensive knowledge of plant species, which is crucial for identifying endangered plants and implementing conservation strategies. Additionally, the research promotes sustainable harvesting techniques, enhancing livelihoods while safeguarding ecosystems. Recognizing local contributions empowers communities and protects their cultural identity for future generations.

Ethnobotanical studies provide useful insights but have limitations like small sample sizes and narrow community focus, which can lead to biased results. Knowledge in this field may change over time due to globalization and climate shifts. It is often shared orally; risking loss without documentation, and language barriers can obstruct communication. For future research, it is suggested to conduct longitudinal studies, integrate technology like GIS to improve the mapping of plant resources and traditional ecological knowledge, leading to more effective resource management strategies, and use interdisciplinary approaches by working with anthropologists, ecologists, and sociologists.

## Figures and Tables

**Table 1:** Priority ranking of therapeutic plants that serve to cure stomachaches.

Medicinal plants	Respondents									
	A	B	C	D	E	F	G	H	Total	Rank
*Croton macrostachyus*	5	1	3	2	4	3	2	1	21	4
*Rumex nervosus*	2	4	4	3	1	5	4	2	25	3
*Ruta chalepensis*	5	4	5	4	3	5	4	3	33	1
*Allium sativum*	4	3	5	5	2	4	3	4	30	2
*Calpurnia aurea*	3	2	1	2	4	3	2	1	18	5

**Table 2:** Comparing herbal remedies used to cure snake bites.

No	Medicinal plants	Respondent									
		A	B	C	D	E	F	G	H	Total	Rank
1	*Allium sativum*	3	4	5	2	3	4	2	2	25	3
2	*Rumex nepalensis*	4	3	5	4	3	5	1	4	28	2
3	*Olea europaea*	4	3	4	4	2	1	2	2	22	4
4	*Calpurnia aurea*	5	4	3	5	5	4	4	2	32	1
5	*Ruta chalepensis*	4	3	3	4	1	2	1	1	19	5
6	*Phytolacca dodecandra*	3	2	2	3	1	2	1	2	16	6

**Table 3:** Informant consensus factor for seven disease categories.

No	Disease categories	No. of species	No. of use citation	ICF
1	Ailments associated with respiratory organs and throat like common cold, cough, asthma, tuberculosis, bronchi	13	49	0.75
2	Dermatology problems like kunchir, skin cuts, wounds, allergy	24	51	0.54
3	Gastrointestinal and abdominal problems like stomachaches, diarrhea, vomiting, ascaris, tapeworm, abdominal pain	28	64	0.57
4	Sexual and delivery problems like syphilis, RH factors, retained placenta	6	8	0.28
5	Organ diseases like eye disease, bone fracture, kidney problems, headaches	14	26	0.48
6	Acute sickness emergency, malaria, hypertension, fever, illness, and evil eye	20	42	0.53
7	Livestock diseases like colic, leech, lice, injuries to the eye, and blackleg.	6	10	0.44

**Table 4:** Fidelity value of traditional therapeutic plants for the ailment that is most commonly reported.

Medicinal plants	Ailments cured	Ni	*N*	Ni/*N*	(Ni/*N*)100
*Allium sativum*	Stomachache	21	25	0.84	84
*Dodonaea angustifolia*		13	15	0.86	86
*Rumex nervosus*		8	9	0.88	88

*Carissa spinarum*	Evil eye	12	12	1	100
*Capparis tomentosa*		9	11	0.81	81
*Pterolobium stellatum*		8	8	1	

*Schrebera alata*	Uvulitis	17	18	0.94	94
*Ruta chalepensis*		20	23	0.86	86

*Ruta chalepensis*	Common cold	6	8	0.75	75
*Allium sativum*		5	5	1	100
*Eucalyptus globulus*		12	13	0.92	92

*Ruta chalepensis*	Acute sickness	23	26	0.88	88
*Capparistomentosa*		7	18	0.38	38

*Dodonaea angustifolia*	Wound	5	6	0.83	83
*Ficus ingens*		6	10	0.6	60
*Laggera tomentosa*		13	24	0.54	54

**Table 5:** List of medicinal plants in the research area that are used to treat human and animal illnesses.

S/no	Botanical name	Family name	Local name	Growth habit	Parts used	Disease treated	Mode of preparation	Route of administration
1	*Acacia abyssinica*	Fabaceae	Girar	Tree	Leaf	Goiter	The tip of a needle is used to break an *Acacia abyssinica* leaves and apply the fluid to the goiter for three consecutive days	Dermal

2	*Allium sativum*	Alliaceae	Nechi shinkurt	Herb	Bulb	Malaria	*Allium sativum* bulbs and ginger rhizomes are mashed and consumed with honey	Oral
					Bulb	Stomach problem	*Allium sativum* bulbs and *Lepidium sativum* seeds are combined and taken with injera	Oral

3	*Buddleja polystachya*	Loganiaceae	Amfar	Tree	Leaf	Eye disease	Livestock eyes are bitten, and *Buddleja polystachya* leaves were chewed and spat out.	Eye

4	*Calpurnia aurea*	Fabaceae	Digita	Shrub	Leaf	Scabies	Livestock with scabies are washed with crushed leaves of *Calpurnia aurea, Croton macrostachyus*, and *Justicia schimperiana*	Dermal
					Leaf	Snake bite	Three to four drops of the crushed *Calpurnia aurea* leaf are administered orally to livestock, while two to three drops are given to humans	Oral
					Leaf	Lumpy skin	Livestock skin is immediately massaged with crushed *Calpurnia aurea* leaves	Dermal

5	*Capparis tomentosa*	Capparidaceae	Gumero	Shrub	Root	Sudden sickness	*Capparis tomentosa* root is kept by drying and powdering it. A person is given one spoonful of the powder combined with alcohol	Oral
					Leaf	Evil eye	*Ruta chalepensis* and *Capparis tomentosa* leaves are crushed and combined with water to make a homemade alcohol cup	Oral
					Root	Intestinal worm	The dry and ground root of *Capparis tomentosa* is combined with water. For three consecutive days, three spoons are given daily	Oral
					Leaf	Tooth infection		Tooth surface

6	*Carissa spinarum*	Apocynaceae	Agam	Shrub	Root	Evil eye	*Carissa spinarum* root is dried out and crushed. The evil eye is treated with dry smoke	Nasal
					Leaf	Headache	*Carissa spinarum* leaves are dried and pulverized. Headaches are treated with dry smoke	Nasal
					Leaf	Stomachache	Pulverized *Carissa spinarum* leaves combined with honey. Especially in the early hours, two to three spoons are taken	Oral
					Root	Gonorrhea	The cool water is combined with freshly crushed *Carissa spinarum* root. For three days, one cup of tella is consumed as a beverage	Oral

7	*Croton macrostachyus*	Euphorbiaceae	Bissana	Tree	Leaf	Ring worm	After crushing and smashing the *Croton macrostachyus* leaf, the resulting substance is applied to the injured region	Dermal
					Bark	Gonorrhea	Three to four spoons of the ground *Croton macrostachyus* and *Vernonia hymenolepis* bark are eaten with tella	Oral
					Leaf	Scabies	Chopped leaves of *Brucea antidysenterica* and *Croton macrostachyus* serve as a calf bodywash	Dermal
					Root	Evil eye	*Carissa spinarum* and *Croton macrostachyus* roots are minced, combined, and disinfected	Nasal
					Leaf	Febrile illness	*Ocimum urticifolium* and *Croton macrostachyus* leaves are treated with fumigation	Oral and nasal
					Leaf	Headache	*Ocimum urticifolium* and *Croton macrostachyus* leaves are crushed and scented	Nasal
					Bark	Wound	*Croton macrostachyus* bark is dried, ground, and used on wounds	Dermal
					Leaf	Infection	Livestock receive mashed *Allium sativum* bulbs and *Croton macrostachyus* leaves	Oral

8	*Clerodendrum myricoides*	Lamiaceae	Misirich	Shrub	Root	Snake bite	The remedy for snake bites is the root	Oral

9	*Dodonaea angustifolia*	Sapindaceae	Kitikita	Shrub	Leaf	Wound	The leaf is used to treat the wound	Dermal

10	*Eucalyptus globulus*	Myrtaceae	Bahir zaf	Tree	Leaf	Avian cholera		Oral
					Leaf	Influenza	*Eucalyptus globulus* leaves are cut and then cooked. People took thermal baths	Nasal

11	*Ficus ingens*	Moraceae	Sesichi	Tree	Sap	Wound	Cattle skin is immediately rubbed with *Ficus ingens* sap	Dermal

12	*Ficus vasta* forssk	Moraceae	Warka	Tree	Sap	Hemorrhoid	*Ficus vasta* fluid and *Pterolobium stellatum* root extract are combined and applied topically to the superficial hemorrhoid	Anal

13	*Grewia ferruginea*	Tiliaceae	Lenquata	Shrub	Latex	Retained placenta	Livestock are given a cup of tella after the resin of Grewia ferruginea is pulverized and combined with water	Oral

14	*Lippia adoensis*	Verbenaceae	Kessie	Shrub	Leaf	Ring worm	*Lippia adoensis* leaves are applied topically to the afflicted area	Dermal

15	*Ocimum gratissimum*	Lamiaceae	Damakesie	Shrub	Leaf	Febrile illness	*Ocimum gratissimum* leaves are broken up and smelt	Nasal

16	*Phytolacca dodecandra*	Phytolaccaceae	Endode	Shrub	Root	Liver problem	The root of phytolacca dodecandra is pulverized and combined with water. A human being is given three percent of a cup	Oral
					Root and leaf	Hyena bite	Livestock are wrapped around the neck with a clean towel after the root of *Phytolacca dodecandra* is broken using the leaf	Neck
					Root	Gonorrhea	Individuals are provided with 1-2 cups of coffee with powdered phytolacca dodecandra and *Croton macrostachyus root*	Oral
					Root	Rabies	One to two cups of domestic alcohol made from the powdered dried root of *Phytolacca dodecandra* are consumed by humans, while three to four cups are used for animals	Oral

17	*Pterolobium Stellatum*	Fabaceae	Kentefa		Root	Evil eye	Root of *Pterolobium stellatum* and root of *Ruta Chalepensis* are powdered and sniffed	Nasal
					Root	Headache	Root of *Pterolobium stellatum* and root of *Ruta Chalepensis* are powdered and sniffed	Nasal

18	*Ricinus communis*	Euphorbiaceae	Gulo	Shrub	Root	Sudden sickness	Cool water is used for grinding the roots of *Ricinus communis* and *Justicia Schimperiana*. Livestock are fed one or two cups of tea	Oral
					Root	Blotting	*Ricinus communis* root mashed with common salt and cool water. Animals receive half a cup	Oral
					Root	Actinomycosis	Soil and common salt are used to pound the root of *Ricinus communis*. Cattle are given a single cup of the mixture, while goats and sheep are given half a glass	Oral
					Fruit	Ulcerative lymphangitis	The donkey's wound skin is treated with a mixture of powdered *Ricinus communis* fruit and *Prunus cuspidata* bark	Dermal
					Fruit	Epizootic lymphangitis	Horse and mule wounds to the skin are treated with a mixture of powdered *Ricinus communis* fruit and *Prunus cuspidata* bark	Dermal
					Fruit	Anthrax	Water is mixed with powdered *Ricinus communis* fruit. A single cup of tea was given to the animals	Oral

19	*Rumex nervosus*	Polygonaceae	Embacho	Shrub	Root	Skin rash (schiffe)	*Rumex nervosus* root is dried and ground into a powder form. Three to four spoonful of the powder are combined with butter and applied to the painful area	Dermal

20	*Ruta chalepensis*	Rutaceae	Tenadam	Shrub	Leaf	Stomachache	A single cup of household alcohol is consumed by the human after the leaves of *Vernonia amygdalina* and *Ruta chalepensis* are crushed altogether	Oral
					Bark and leaf	Coccidiosis	*Justicia schimperiana* root, *Ruta chalepensis* bark, and leaves are crushed mixed and fed to hens with injera	Oral
					Leaf	Cough	*Cussonia Ostinii chiovof* is used to pound *Ruta chalepensis* leaves, which are then consumed with injera	Oral
					Leaf	Influenza	*Ruta chalepensis* leaves are mashed with *Allium sativum* bulbs and combined with soup to make a beverage	Oral

21	*Schinus molle*	Anacardiaceae	Qundoberibere	Tree	Leaf and fruit	Eye disease	Livestock, horses, goats, and sheep's eyes are bitten and spat on *Schinus molle* leaves and fruit	Optical

22	*Schrebera alata*	Oleaceae	Sembo	Tree	Leaf	Tonsillitis	Tonsillitis chews and spit out a *Schrebera alata* leaf	Oral

23	*Thymus schimperi*	Lamiaceae	Tosigni	Shrub	Whole parts	Blood pressure	The entire section is utilized to treat hypertension	Oral

24	*Dodonaea angustifolia*	Sapindaceae	Kitikita	Shrub	Leaf	Eczema (schiffe)	The entire section is utilized to treat hypertension	Dermal

25	*Olea europaea* L*.subsp. cuspidate*	Oleaceae	Woyira	Tree	Leaf	Eye disease	*Olea europaea* leaves are crushed, ground into a powder, and inserted via the eye	Optical

26	*Osyris quadripartita*	Santalaceae	Kereti	Shrub	Root	Evil eye	The roots of the plant and *Carissa spinarum* roots are pounded, dried, and burned, and the resulting smoke is inhaled	Nasal
					Leaf	Skin infection	The beaten leaves were combined with water and applied to the afflicted skin	Dermal

27	*Otostegia integrifolia*	Lamiaceae	Tinjut	Shrub	Leaf and stem	Evil sprite	Plant stem and leaf were smashed and set it on fire, and the smoke was inhaled.	Nasal

28	*Hibiscus micranthus* L.f	Malvaceae	Nechilo	Shrub	Leaf and fruit	Eye disease	Cattle, horses, goats, and sheep's eyes are bitten and spat on *Schinus molle* leaves and fruit	Optical

29	*Jasminum abyssinicum* hochst. Ex DC	Oleaceae	Tenbelel	Shrub	Leaf	Tonsillitis	Tonsillitis chew and throw out a *Schrebera alata* leaf	Oral

30	*Jasminum grandiflorum* L.	Oleaceae	Qega	Shrub	Whole parts	Blood pressure	Whole part used to treat blood pressure	Oral

31	*Juniperus procera* Hochst. Ex Endl		Yehabesha tsidie	Tree	Leaf	Eczema (schiffe)	Whole part used to treat blood pressure	Dermal

32		Cupressaceae			Leaf	Eye disease	The plant's root and the *Carissa spinarum* root are struck and dried out, and set on fire	Optical

33	*Justicia schimperiana* (Hochst. Ex Nees) T Anders	Acanthaceae	Sensel	Shrub	Root	Evil eye	The remedy for snake bites is the root	Nasal

34	*Maytenus arbutifolia* (A. Rich.) Wilczek	Celastraceae	Atat	Shrub	Root	Snake bite	The smoke is then inhaled after the crushed, powdered leaf of *Olea europaea* is inserted via the eye	Oral

35	*Maytenus senegalensis* (Lam.) Exell	Celastraceae	Furkita	Shrub	Leaf	Wound	The leaf was used to treat the wound	Dermal

36	*Melia azedarach* L.	Meliaceae	Neem	Tree	Leaf	Avian cholera		Oral

37	*Myrsine Africana* L.	Myrsinaceae	Kechemo	Shrub	Leaf	Influenza	Leaf of *Eucalyptus globulus* is chopped and boiled. Steam bath is taken by human	Nasal

38	*Ocimum gratissimum* L.	Lamiaceae	Besobila	Herb	Sap	Wound	Sap of *Ficus ingens* is directly creamed on cattle skin	Dermal

39	*Olea europaea* L.subsp. uspidate (Wall. Ex G.Don) Cif	Oleaceae	Woyira	Tree	Sap	Hemorrhoid.	Sap from *Ficus vasta* and powdered root of *Pterolobium stellatum* are mixed together and creamed topically to the external hemorrhoid	Anal

40	*Osyris quadripartite* Decn.	Santalaceae	Kereti	Shrub	Root	Snake bite	The root was used to treat snake bite	Oral

41	*Ocimum lamiifolium Benth*.	Lamiaceae	Demakesie	Shrub	Leaf	Wound	The leaf was used to treat the wound	Dermal

42	*Otostegia integrifolia* benth	Lamiaceae	Tinjut	Shrub	Leaf	Avian cholera		Oral

43	*Phytolacca dodecandra* L'Herit	Phytolaccaceae	Endode	Shrub	Leaf	Influenza	Leaf of *Eucalyptus globulus* is chopped and boiled. Steam bath is taken by human	Nasal

44	*Prunus Africana (Hook. F.) Kalkam*	Rosaceae	Tikur inchet	Tree	Sap	Wound	Sap of *Ficus ingens* is directly creamed on cattle skin	Dermal

45	*Pterolobium stellatum* (forssk) brenan	Fabaceae	Kentefa	Shrub	Sap	Hemorrhoid.	*Ficus vasta* sap and *Pterolobium stellatum* root powder are combined and applied topically to the external hemorrhoid	Anal

46	*Podocarpus falcatus*	Podocarpaceae	Zigiba	Tree	Latex	Retained placenta	Cattle receive a cup of tella after the latex of *Grewia ferruginea* is pulverized and combined with water	Oral

47	*Rhus glutinosa A.Rich*	Anacardiaceae	Embis	Shrub	Leaf	Ring worm	*Lippia adoensis* leaves are applied topically to the afflicted area	Dermal

48	*Rhus natalensis* krauss	Anacardiaceae	Takima	Tree	Leaf	Febrile illness	*Lippia adoensis* leaves are applied topically to the afflicted area	Nasal

49	*Rhus retinorrhoea* Oliv.	Anacardiaceae	Talo	Tree	Root	Snake bite	The root was used to treat snake bite	Oral

50	*Ricinus communis* L.	Euphorbiaceae	Agulo	Shrub	Root	Snake bite	The root was used to treat snake bite	Oral

51	*Rhamnus Prinoides* L*'Herit*.	Rhamnaceae	Gesho	Shrub	Leaf	Wound	The leaf was used to treat the wound	Dermal

52	*Rumex nervosus* Vahl	Polygonaceae	Embacho	Shrub	Leaf	Avian cholera	*Lippia adoensis* leaves are applied topically to the afflicted area	Oral

53	*R. chalepensis* L.	Rutaceae	Tenadam	Shrub	Leaf	Influenza	*Eucalyptus globulus* leaves are cut and then cooked. People take steam baths	Nasal

54	*Rumex abyssinicus Jacq.*	Polygonaceae	Mekimeko	Herb	Sap	Wound	Livestock skin is gently rubbed with *Ficus ingens* sap	Dermal

55	*Rumex nepalensis*	Polygonaceae	Tulit	Herb	Sap	Hemorrhoid.	*Ficus vasta* sap and *Pterolobium stellatum* root powder are combined and applied topically to the external hemorrhoid	Anal

56	*Sansevieria forskaliana* (Schult.f) Hepper and wood	Dracaenaceae	Cheret	Shrub	Latex	Retained placenta	Cattle are given a glass of tella after the waxy substance of *Grewia ferruginea* is pulverized and combined with water	Oral

57	*Hibiscus micranthus* L.f	Malvaceae	Nechilo	Shrub	Leaf and fruit	Eye disease	Animals, horses, goats, and sheep's eyes are bitten and *Schinus molle* leaves and berries were chewed and spat out.	Optical

58	*Solanum tuberosum*	Solanaceae	Dinich	Herb	Leaf	Tonsillitis	Tonsillitis consume and spit out a *Schrebera alata* leaf	Oral

59	*Citrus medica*	Rutaceae	Tirgo	Shrub	Whole parts	Blood pressure	Whole part was used to treat blood pressure	Oral

60	*Citrus lemon*	Rutaceae	Lomi	Shrub	Leaf	Eczema (schiffe)	Whole part was used to treat blood pressure	Dermal

61	*Citrus sinensis*	Rutaceae	Birtucan	Shrub	Leaf	Eye disease	The plant's root and the *Carissa spinarum* root are banged dried, and set on fire	Optical

62	*Lycopersicon esculentum*	Solanaceae	Tematim	Herb	Root	Evil eye	The beaten leaves were combined with water and applied to the afflicted skin	Nasal

63	*Saccharum officinarum*	Poaceae		Herb	Leaf	Skin infection	The smoke is then inhaled after the crushed, powdered leaf of *Olea europaea* is added through the eye	Dermal

64	*Laggera tomentosa* (sch. Bip. Ex A. Rich.) Olivo & hiern	Asteraceae	Alashumie	Shrub	Leaf and stem	Evil sprite	The stem and leaf of the plant were crushed and put on the fire and the smoke was inhaled.	Nasal

65	*Lepidium sativum*	Brassicaceae	Feto		Leaf and fruit	Eye disease	Cattle, horses, goats, and sheep's eyes are bitten, and *Schinus molle* leaves and berries were chewed and spat out.	Optical

66	*Lippia adoensis* Hochst. Ex Walp	Verbenaceae	Kessie	Shrub	Leaf	Tonsillitis	Tonsillitis bite and spit out a *Schrebera alata* leaf	Oral

67	*Cucurbitaceae pop*	Cucurbitaceae	Duba		Leaf and fruit	Eye disease	Livestock, horses, goats, and sheep's eyes are bitten, and *Schinus molle* leaves and berries were chewed and spat out.	Optical

68	*Ximenia Americana* L.	Oleaceae	Enkoy	Shrub	Leaf	Tonsillitis	Tonsillitis consumes and spit out a *Schrebera alata* leaf	Oral

69	*Urtica simensis Steudel*	Urticaceae	Sama	Herb	Whole parts	Blood pressure	The entire section is utilized to treat hypertension	Oral

70	*Verbena officinalis* L.	Verbenaceae	a	Herb	Leaf	Eczema (schiffe)	The entire section is utilized to treat hypertension	Dermal

71	*Ziziphus spinachristi* (L.) Desf	Rhamnaceae	Qurqura	Shrub	Leaf	Eye disease	Through the eye, the ground and pulverized leaf of *Olea europaea* is introduced	Optical

72	*Eucalyptus globulus*	Myrtaceae	Bahir zaf	Tree	Root	Evil eye	The plant's and *Carissa spinarum's* roots were mashed, dried, and burned; the smoke was then inhaled	Nasal

73	*Laggera tomentosa* (Sch. Bip. Ex A. Rich.) Olivo & Hiern	Asteraceae	Alashumie	Shrub	Leaf	Skin infection	The beaten leaves were combined with water and applied to the afflicted skin	Dermal

74	*Lepidium sativum*	Brassicaceae	Feto		Leaf and stem	Evil sprite	Plant stem and leaf were mashed and set it on fire, and the smoke was inhaled.	Nasal

**Table 6:** The percentage of every type of plant used to cure cattle illnesses that are most common in the research site.

S/no	English name	Common name	No. of plant species	Percentage of all medicinal plants utilized by animals
1	Anthrax	Quriba	2	14
2	Anti-inflammatory	Yesewonti mabeti	3	21
3	Avian cholera	Teqimat	1	7
4	Blackleg		1	7
5	Blotting	Yekoda beshita	1	7
6	Cough	Sali	2	14
7	Diarrhea	Teqimati	3	21
8	External parasites	Yewichi tigegna	3	21
9	Eye problem	Eyine himem	2	14
10	Foot-and-mouth diseases	Afemazi	1	7
11	Hyena bite	Yegib nikisha	1	7
12	Infection	Qusilet	1	7
13	Internal parasites	Yewisit tigegna	3	21
14	Leeches	Qimal	1	7
15	Retained placenta	Engideliji	2	14
16	Wound	Qusil	3	21

**Table 7:** Human diseases that are common in the research site and the percentage of plant species utilized to cure each condition.

S/no	Illness cured	Local name	The number of plant species	Proportion of all herbal remedies utilized by individuals
1	Amoeba case	Amoeba	3	6.25
2	Ascaris	Yemnteko tili	2	4.1
3	Back pain	Yegerba himem	1	2
4	Cough	Gunfan	4	8.3
5	Diarrhea	Teqimati	5	10.4
6	Ear pain	Yejoro himmi	2	4.1
7	Epilepsy	Yemetil beshita	3	6.25
8	Evil eye	Buda	2	4.1
9	Eye infection	Eyin beshita	3	6.25
10	Fire burn	Mekatli	2	4.1
11	Gonorrhea	Chebiti	1	2
12	Headache	Ras mitat	3	6.25
13	Hepatitis	Yewofi beshita	2	4.1
14	Snake bite	Ebab menedefi	2	4.1
15	Spider poison	Yshererti merizi	1	2
16	Stomach problem	Yehode himem	9	18.8
17	Sudden sickness	Dinigetegna	3	6.25
18	Tapeworm	Yekoso til	2	4.1
19	Teeth infection	Yetirisi himem	3	6.25
20	Tetanus	Tetanesi	3	6.25
21	Tonsil	Entil mewiredi	4	8.3
22	Wound	Qusile	5	10.4
23	Excessive bleeding	Demi mefisesi	4	8.3
24	Evil sprite	Aganit	3	6.25
25	Nasal bleeding	Nesir	2	4.1
26	Hemorrhoids	Kintarot	3	6.25
27	Rabies	Yewsha beshita	4	8.3
28	Skin rash	Qoda masakeki	2	4.1

**Table 8:** The average number of herbal remedies reported in the research area and the participants' chronological age.

Age range	Midpoint (*x*)	Average med. plant cited (*Y*)	*XY*	*X* ^2^	*Y* ^2^
18–29	23.5	9	211.5	552.5	81
30–45	37.5	11	412.5	1406.25	121
46–60	53	14	742	2809	196
61–80	70.5	22	1551	4970.5	484
Total	184.5	56	2917	9738.25	882

**Table 9:** Ordering of risks to therapeutic plants.

	Major risk	Respondents								Total	Rank
		A	B	C	D	E	F	G	H		
1	Farm land expansion	5	4	4	5	3	5	4	3	33	1
2	Drought	2	1	3	4	4	2	3	3	22	3
3	Construction material	1	2	2	1	3	1	2	2	14	4
4	Charcoal making	4	4	3	3	3	1	3	1	22	3
5	Road construction	3	4	3	3	3	4	5	4	29	2

## Data Availability

Data used to support the findings of this study are included within the article. No separate data are available anywhere, but the authors included all the data under the text.
